# Feeding Fiber-Bound Polyphenol Ingredients at Different Levels Modulates Colonic Postbiotics to Improve Gut Health in Dogs

**DOI:** 10.3390/ani12050627

**Published:** 2022-03-02

**Authors:** Dennis E. Jewell, Matthew I. Jackson, Chun-Yen Cochrane, Dayakar V. Badri

**Affiliations:** 1 Department of Grain Science and Industry, Kansas State University, Manhattan, KS 66506, USA; djewell@ksu.edu; 2 Hill’s Pet Nutrition, Inc., Topeka, KS 66617, USA; matthew_jackson@hillspet.com (M.I.J.); chun-yen_cochrane@hillspet.com (C.-Y.C.)

**Keywords:** canine, fiber bundle, bioactive metabolites, microbiota, anti-inflammatory, short-chain fatty acids

## Abstract

**Simple Summary:**

Microbes present in the large intestine of humans and companion animals produce bioactive metabolites from host-ingested food. These bioactive metabolites can influence host health. A prior study in dogs that were healthy or had chronic enteritis/gastroenteritis showed that stool quality improved when they ate food containing a fiber bundle made from fibers of pecan shells, flax seed, cranberry, citrus, and beet. In addition, eating food containing the fiber bundle resulted in the gut bacteria shifting from digesting mainly protein to digesting mainly carbohydrates. The present study tested the impact of the fiber bundle at a lower range of concentrations in dogs. Fecal levels of several bioactive metabolites with beneficial antioxidant or anti-inflammatory properties increased after dogs consumed food with the fiber bundle, though no changes in the bacteria or their functional pathways were observed. Stool quality remained in the acceptable range. These results suggest that the gut bacteria were able to digest the fiber bundle to produce beneficial bioactive metabolites to improve host health.

**Abstract:**

This study assessed changes in canine fecal metabolites and microbiota with the consumption of foods with increasing concentrations of a fiber bundle including pecan shells, flax seed, and powders of cranberry, citrus, and beet that was previously shown (at 14% *w*/*w*) to improve stool quality, shift fecal bacterial metabolism from proteolysis to saccharolysis, increase abundance of saccharolytic bacteria, and decrease abundance of proteolytic bacteria. In this study, 48 healthy adult dogs were split evenly to consume different inclusion levels (0%, 1%, 2%, and 4%) of the fiber bundle for a 31-day period following a 28-day pre-feed period. Increases from baseline in the fecal short-chain fatty acids butyric acid, valeric acid, and hexanoic acid were observed only in the dogs that consumed the food with the 4% fiber bundle. With addition of any level of the fiber bundle, increases were seen in the polyphenols hesperidin, hesperetin, ponciretin, secoisolariciresinol diglucoside, secoisolariciresinol, and enterodiol. However, fecal microbiota and their metabolism, and stool scores were largely unaffected by the fiber bundle. Overall, addition of the fiber bundle appeared to increase bioactive metabolites of increased antioxidant and anti-inflammatory potency for beneficial to health and, at levels ≥4%, shifted gut bacterial metabolism toward saccharolysis.

## 1. Introduction

The interplay among nutrition, the gut microbiota and its metabolites, and host health has been the subject of numerous studies over the last decade [1,2]. Dietary consumption of fiber is known to yield health benefits, and many fiber-rich foods are also high in polyphenols. Most dietary polyphenols arrive largely intact to the large intestine, where they are metabolized by intestinal microbiota [3], and these postbiotic compounds are proposed to confer many of the observed beneficial health effects [4]. Further, these compounds appear to modulate the gut microbiota, with a decrease in pathogenic bacteria [4,5].

Consumption of high-fiber foods also results in the increase of short-chain fatty acids (SCFAs), which are used as energy sources for colonocytes, contribute to maintenance of the intestinal tight junction barrier, and decrease inflammation [6,7]. An increase in SCFAs is also considered to be beneficial since they are decreased in people with ulcerative colitis or Crohn disease [8].

Several studies have examined the role of various added fibers in canine food in changing the gut microbiota and/or metabolites [9,10,11,12,13,14,15,16]. One of these prior studies tested a fiber bundle composed of pecan shells, flax seed, and cranberry, citrus, and beet powders to provide lignin as an insoluble bulking fiber, moderately fermentable fibers (whole flax), and fermentable fibers (e.g., hemicellulose and pectin) [16]. The change in fecal metabolites with the addition of the fiber bundle appeared to shift metabolism from a proteolytic state toward saccharolysis when added to either hydrolyzed meat or grain-rich foods in dogs that were healthy or had chronic enteritis/gastroenteritis [16]. In addition, the abundance of saccharolytic bacteria increased and proteolytic bacteria decreased with inclusion of the fiber bundle. Stool quality [17] also improved in both healthy dogs and those with chronic enteritis/gastroenteritis following consumption of food with the fiber bundle [16].The present study extended the original study by testing the fiber-bound polyphenolic ingredients of the fiber bundle at different levels in a single type of canine food (grain-rich), with the purpose of testing the differential impact on fecal metabolites and microbiota as availability of substrates for fermentation decreased.

## 2. Materials and Methods

### 2.1. Study Foods

All foods were manufactured at the Hill’s experimental food laboratory. The control food used in this study was the same as the grain-rich control food used in a previous study [16]. The test foods were of similar formulation to the control food except they were supplemented with the fiber bundle (composed of ground pecan shells, dried pelleted beet pulp, ground citrus pulp, whole brown flax seed, and cranberry pomace) at 1%, 2%, or 4% *w*/*w* on a dry matter basis and replaced all or some of the cellulose in the control food. Compositions of the foods, all in dry form, were formulated to canine adult maintenance standards and were equivalent across control and test foods (Table 1) to ensure that any differences observed were due to the addition of the fiber bundle rather than perturbation of macronutrients. Food formulations are shown in Table S1. All foods met the maintenance nutrition requirements of the Association of American Feed Control Officials.

### 2.2. Animals and Experimental Design

The study protocol was approved by the Hill’s Institutional Animal Care and Use Committee (IACUC; CP848a.0.0.0-A-C-D-ADH-MULTI-98-GI). This study complied with the guide for the care and use of laboratory animals from the US National Research Council [18].

**Figure 1:**
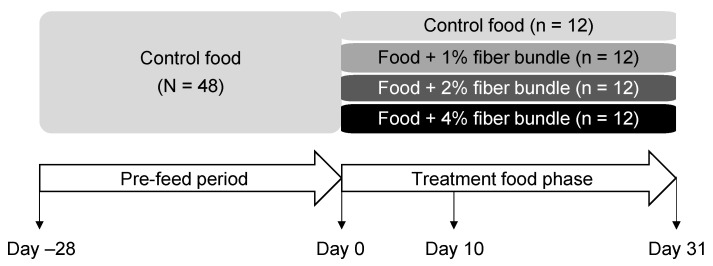
Study design and timeline in which dogs consumed foods containing 0%, 1%, 2%, or 4% of the fiber bundle for 31 days. Blood and fecal samples were collected at days 0, 10, and 31 of the treatment food phase.

A total of 48 healthy adult dogs (24 female, 24 male), all spayed or neutered and owned by Hill’s Pet Nutrition, Inc., were included. Dogs were housed at the Pet Nutrition Center in pairs with regular access to natural light, daily exercise, and socialization opportunities. All dogs were fed the control food for 28 days and then divided into four groups of 12 dogs each based on their sex, age, and body weight (Figure 1). Each group continued into the treatment phase in a parallel experimental design with consumption of the control food or foods with the fiber bundle added to 1%, 2%, or 4% for 31 days. Feeding each dog the control food prior to the treatment food phase should reduce the individual effect in the later microbiota analysis. Each dog was fed based on their caloric requirements as calculated by their body weight. All dogs were healthy at the end of the study, with no adverse events reported.

Blood/serum and feces from each dog were collected at the end of pre-feed phase and at days 10 and 31 days in the treatment phase. Dogs were sedated before phlebotomy. After collecting the blood samples in serum separator tubes (SST™ Serum Separation Tubes, Fisher Scientific, Waltham, MA, USA), samples remained at room temperature for 20 min to clot and were then centrifuged for 10 min at room temperature to separate serum from blood cells. Collected serum was aliquoted and stored at −70 °C for various analyses. One fecal sample per dog was collected within 30 min of defecation.

### 2.3. Stool Scoring and Fecal Sample Processing

Fecal scores were determined on a 1–5 scale where grade 1: no solid form and >75% liquid, grade 2: soft and 50% solid and 50% liquid, grade 3: some cylindrical shape and >75% formed and solid, grade 4: >75% cylindrical and >50% firm, and grade 5: cylindrically shaped and >80% firm [17]. Each fecal sample was then homogenized using a Thinky Mixer (Thinky USA, Inc., Laguna Hills, CA, USA) following the Hill’s Pet Nutrition protocol as previously described [16]. Fecal pH was measured immediately after homogenization. Homogenized fecal samples were frozen at −70 °C until analysis.

### 2.4. Serum and Metabolite Analyses

Blood count profiles (Sysmex XN 1000-V, Sysmex America, Inc., Lincolnshire, IL, USA) and serum chemistry (Cobas c501, Roche Diagnostics, Indianapolis, IN, USA) were analyzed according to manufacturers’ instructions. Fecal SCFAs and metabolites were analyzed by Metabolon, Inc. (Morrisville, NC, USA).

### 2.5. Fecal Microbiome Analysis and Bioinformatics Processing

Fecal microbiome analysis was performed utilizing the Hill’s Pet Nutrition protocols [19]. Briefly, total DNA was extracted from frozen feces samples using the PowerFecal DNA isolation kit (MO BIO, Carlsbad, CA, USA) according to the manufacturer’s instructions with the modification of introducing a sonication step before vortexing the bead tubes with fecal samples horizontally for 15 min. PCR amplification used the primer pairs spanning the V3–V4 hypervariable regions of the 16S rRNA gene. Sequencing was performed using the Illumina (San Diego, CA, USA) MiSeq platform, and the resulting sequences were de-multiplexed based on the dual index sequences by employing the MiSeq built-in metagenomics workflow to obtain FASTQ files. FASTQ sequence files were processed using the standard parameters of the Mothur software [20] and taxonomic classification was obtained by using the Greengenes reference database [21]. Operational taxonomic units (OTUs) were identified based on taxonomic hierarchy and further processed using the Phylogenetic Investigation of Communities by Reconstruction of Unobserved States (PICRUSt) protocol [22] to correct for copy numbers of the 16S genes in their respective taxa, followed by predicting functional attributes using the Kyoto Encyclopedia of Genes and Genomes (KEGG) database.

### 2.6. Statistical Analysis

Fecal microbiome data were analyzed at the phylum, family, and genera levels. Only the OTUs and the PICRUSt-predicted KEGG ortholog functions present in at least 70% of all the fecal samples were considered in the following statistical analysis. The individual OTU counts (corrected for 16S copy number) were analyzed by a negative binomial mixed model to study the effects of food treatments and collection time-points. The model also included a pre-feed covariate and a random animal factor. Permutational multivariate analysis of variance (PERMANOVA) based on the Manhattan distance of relative abundances was used to compare microbial compositions and custom-curated KEGG orthlog functional compositions among food treatments. All *p* values were adjusted for false discovery rate (FDR) using the Benjamini–Hochberg procedure. A principal coordinate analysis plot was made to visualize the proximity of the four food treatments on Day 31 using the Manhattan distance of the fecal microbial composition at the genus level.

For the metabolite analysis, all values were transformed to natural logs and the change was calculated by subtracting initial values from final values. Mean differences and change over time were evaluated by the Proc Mixed procedure of SAS 9.4. Values represented for fecal metabolites are in relative fold differences. Unlike the absolute values presented for other endpoints (e.g., SCFA), which can be compared to previously published reports, relative fold differences in metabolomics have meaning only in terms of change or differences of responsiveness between groups. Thus, fecal metabolite analyses report the significance of change from baseline (Day 0) and the degree to which those changes were significantly different among treatment groups.

## 3. Results

### 3.1. Animal Demographics, Intakes, and Clinical Assessment

Of the 48 healthy adult dogs in this study, the mean ± SD age was 4.9 ± 2.3 years (range, 1.2–8.6 years). The mean body weight at baseline was 10.6 ± 1.8 kg (range, 7.7–14.5 kg) and was similar among all groups (Table 2). All dogs were fed the control food for 28 days prior to being divided evenly into four groups that were fed the control food or test foods containing 1%, 2%, or 4% of the fiber bundle for 31 days. Food intake was similar among groups (Table 2). After 31 days of the food treatment period, slightly greater weight loss from baseline was observed in the control and 1% fiber bundle groups but not in the 2% and 4% fiber bundle groups (−0.3 kg and −0.1 kg; Table 2). Serum albumin, urea nitrogen, triglycerides, and cholesterol were similar among groups, with no significant differences from baseline or among groups. A significant decrease was seen in serum total protein in the 1% group compared with baseline, as were significant increases from baseline in creatinine in the 2% and 4% groups. However, levels of these circulating markers remained within clinically normal ranges.

### 3.2. Fecal Parameters and Analytes

Fecal moisture following consumption of the test foods significantly differed among groups at 31 days and differed from baseline within some individual foods, but these small changes were not considered to be physiologically relevant (Table 3). Fecal ammonium levels and pH were similar among all groups at baseline and showed no notable changes at the end of the 31-day feeding period. Stool scores were within acceptable ranges and showed no significant differences at any time points within the study.

Fecal SCFAs were assessed at baseline and days 10 and 31 of the feeding treatment phase. The straight-chain SCFAs butyric acid, valeric acid, and hexanoic acid were all significantly greater than baseline in feces from dogs in the 4% fiber bundle group (Table 4). Otherwise, the control groups and those with lower levels of the fiber bundle did not show significant changes in straight-chain or branched-chain SCFAs among groups or from their baseline values.

The concentrations of several dietary plant compounds and their postbiotics were significantly increased in feces from dogs fed any of the tested levels of the fiber bundle (Table 5). These included the polyphenols hesperidin, hesperetin, ponciretin, secoisolariciresinol diglucoside (SDG), secoisolariciresinol, and enterodiol. Arabinose was significantly increased in feces from the group that consumed the 4% fiber bundle food at both the day 10 and day 31 timepoints, while ribulose/xylulose was significantly increased in that group at day 10.

### 3.3. Fecal Microbiota

**Figure 2:**
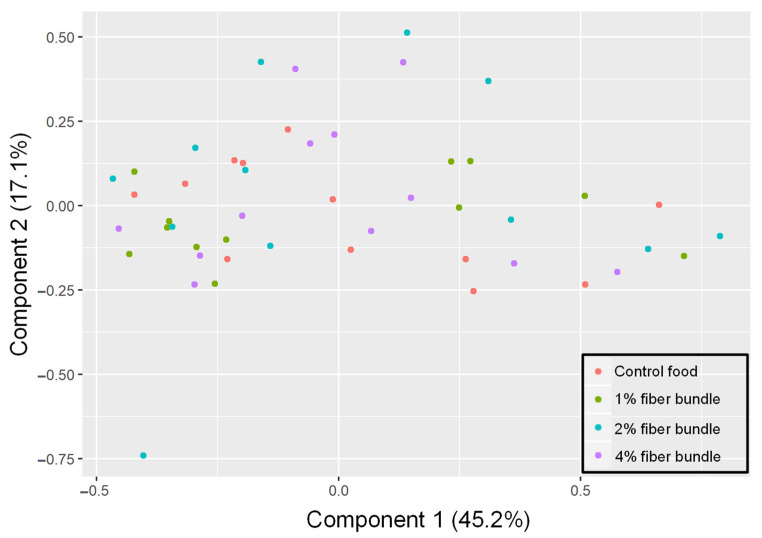
Principal coordinate analysis plot of the fecal microbiome of dogs that consumed foods containing 0%, 1%, 2%, or 4% of added fiber-bound polyphenol ingredients for 31 days.

The principal coordinate analysis plot showed no clear separation in the fecal microbiome among any of the food types studied (Figure 2). Further, PERMANOVA analysis of the fecal microbiome composition at the phylum, family, and genera levels showed no significant differences among groups, nor did the curated KEGG ortholog function compositions (Table 6).

## 4. Discussion

A previous study demonstrated that inclusion of a fiber bundle at 14% in canine food significantly increased the abundance of saccharolytic gut bacteria, decreased the abundance of proteolytic bacteria, and also shifted gut microbial metabolism toward saccharolytic activity [16]. In the current study, we tested the same fiber bundle at various inclusion levels ranging from 1 to 4% while retaining the same ratios of fibrous ingredients in order to determine the lowest inclusion level that would manifest molecular signatures to potentially improve pet health. The inclusion of a fiber bundle in food appeared to lead to a shift toward saccharolytic metabolism of the gut microbiome of dogs fed the 4% fiber bundle food compared to 1% and 2%, as indicated by the increased fecal levels of the SCFA butyrate and the sugar arabinose, which also suggests that gut microbiota could utilize the hemicellulose component of the fiber source. A number of other compounds with antioxidant and anti-inflammatory properties were present at greater levels in feces from dogs that consumed foods with any of the tested levels of the fiber bundle, consistent with the prior study in which the fiber bundle was included at 14% [16]. Thus, although no significant changes in the composition of the gut microbiota were observed in this study, the results indicate that the fiber bundle led to a shift in their metabolism toward the production of beneficial compounds and potentially greater gut health.

It has been previously proposed that the interaction of polyphenols from food and the gut microbiota may positively influence host health [3]. Consumption, in humans, of flavonoids, a subset of polyphenols, has been shown to lower the risk of cancer, cardiovascular diseases, metabolic diseases, and neurodegenerative diseases [23,24]. In human trials, consumption of orange juice, which is rich in flavonoids, appeared to improve cognitive function in adults, and consumption of citrus fruits was inversely correlated with the risk of dementia in elderly participants [23].

Hesperidin, a flavonoid found in citrus fruits, was significantly increased with addition of the fiber bundle at all of the tested concentrations in this study. Supplementation with hesperidin has shown glucose-lowering effects and improvements in insulin resistance and inflammatory parameters in rodent models of both type 1 and type 2 diabetes [25,26]. Other cardiovascular benefits of hesperidin include its observed effects on lipid profiles, hypertension, oxidative stress, and inflammation [25,26]. The health benefits of hesperidin are attributed to its anti-inflammatory effects on cytokines and its antioxidant effects [26].

Hesperetin, which also significantly increased in feces following consumption of food with the fiber bundle, is metabolized from hesperidin by intestinal bacteria [27]. Hesperetin appears to have even greater bioavailability and anti-inflammatory and antioxidant properties than its precursor [26]. These benefits extend to diabetes, metabolic disorder, and other cardiovascular risk factors [25].

Hesperidin and hesperetin have both been observed to confer protective properties on neurons in both in vitro and animal models of various neurological diseases such as Alzheimer disease, epilepsy, Huntington disease, and Parkinson disease, in which cytotoxicity was induced by inflammatory stimuli, oxidative stress, or neurotoxic stress in animal models [23,28]. In addition, hesperetin supplementation led to decreased oxidative stress, inflammation, and apoptosis in a murine model of myocardial ischemia [29]. Several flavonoids, including hesperetin, appear to increase the barrier integrity of the tight junction in human intestinal Caco-2 cells [30]. Extending this to an organismal level, several experiments in rodent models of chemically induced colitis have shown that hesperidin and hesperetin led to improvements in colitis symptoms, including inflammation and colonic barrier function [24].

Hesperetin, hesperidin, and other polyphenols in orange juice appear to stimulate the growth of beneficial bacteria and inhibit pathogenic bacteria in vitro [31,32]. Although a shift in the microbiome was not observed in the present study, this could have been due to the relatively low levels of inclusion of the fiber bundle. In the prior study that examined the effects of the fiber bundle at 14%, the abundances of several genera of saccharolytic bacteria increased while those of potentially pathogenic bacteria decreased following consumption of the fiber bundle in either grain-rich or high-meat-food backgrounds [16].

Another flavonoid, ponciretin, is metabolized from poncirin, derived from citrus fruit, by intestinal bacteria, as shown in humans [27] and mice [33]. Like hesperidin, ponciretin has shown anticancer and anti-inflammatory effects, with cytotoxic effects on a colon cancer cell line in vitro [27] and attenuating colitis in mice by the anti-inflammatory effects of suppressing NF-κB activation and correcting the imbalance of Th17/Treg cells [33,34]. In addition, ponciretin can inhibit the growth of *Helicobacter pylori* [35], a bacterium implicated in gastritis, stomach ulcers, and lymphomas of the gastrointestinal tract [36].

The biological activity of SDG, the main lignan in flaxseed [37], results from its conversion to secoisolariciresinol, then enterodiol, and enterolactone, carried out by intestinal microbiota [38,39]. As in a prior study that tested the addition of low or high soluble fiber with betaine to food in canines, higher fecal levels of SDG and secoisolariciresinol were observed with increasing fiber consumption [40], as was enterodiol. Similar to polyphenols, the antioxidant and anti-inflammatory properties of SDG appear to translate to a number of positive health benefits in a multitude of diseases, including diabetes, cancer, and cardiovascular disease [37,38]. SDG exhibited positive effects in a variety of model systems, including antioxidant effects in cadmium-induced renal toxicity in rats [41], anti-inflammatory effects in the dextran sulfate sodium salt-induced colitis mouse model [42], and cytoprotective and anti-inflammatory effects in human umbilical vein endothelial cells [43]. SDG and enterodiol have shown apoptotic effects on cultured human colon carcinoma cells [44] and colorectal cancer cells in vitro [45], respectively.

The increases of the pentoses arabinose and ribulose/xylulose with the 4% fiber bundle food may have originated from breakdown of the dietary fiber. These sugars can then be fermented by the gut microbiota to produce SCFAs [46], which may also explain the increases in SCFAs observed with the 4% fiber bundle food in this study.

Several of the results seen here are consistent with those of other studies that have studied the effects of consumption of fiber and/or lignans. Increased butyric acid and arabinose have been observed with consumption of fiber in dogs compared with control foods [15,16,47] and indicate a shift from proteolytic to saccharolytic metabolism. Dietary intake of lignans was also associated with higher levels of butyric acid in a human trial [48].

Interestingly, neither the fecal microbiome nor its functional pathways appeared to change to a significant extent with the addition of the fiber bundle in the present study, despite the changes to the metabolome. A recent review noted that food-induced changes in the microbiome of healthy dogs are not as extensive as the microbiome changes observed in disease states [49]. Since the compositions of the gut microbiota clearly shifted in the prior study in which the fiber bundle was included to 14% [16], there may be some minimum level required above the 4% used as the highest level in this study to observe changes in the microbiota. However, it is of interest that the metabolic signatures of the gut microbiome activity were altered at levels of fiber that were too low to induce measurable changes in the genetic signature of that same microbiome.

A limitation of this study was that levels of the fiber bundle were perhaps too low to observe the greater shifts in metabolites and microbiota observed in the previous study with the fiber bundle included at 14% [16]. In addition, it is possible that greater effects would have been seen in the present study if the time had been extended beyond the 31-day feeding period, if more dogs per treatment group were included, or if dogs with an underlying condition, such as enteritis or gastroenteritis, were included. Further studies are needed to determine the optimal levels of different types of fiber for the production of beneficial metabolites to improve pet health.

## 5. Conclusions

In this study, a fiber bundle included in canine food at levels ranging from 1 to 4% was examined for its effects on fecal metabolites and microbiota in healthy dogs. There was virtually no effect on the microbiota at the end of the 31-day feeding period, in contrast to results from a prior study in which the fiber bundle was included at 14%. However, levels of several metabolites with antioxidant and anti-inflammatory properties, including polyphenols and lignans, were increased with addition of the fiber bundle at 4%, as were butyric acid and arabinose. Together, these indicate that the gut microbiota were able to utilize the fiber bundle toward saccharolytic metabolism and transform fiber-bound polyphenols into bioactive metabolites that increased the antioxidant and anti-inflammatory potency for a healthier state.

## Figures and Tables

**Table 1:** Composition of test foods containing 0, 1, 2, or 4% added fiber-bound polyphenol ingredients (grams/100 grams as mixed or as fed, unless otherwise stated).

		Fiber Bundle Percentage in Food		
Nutrient Parameter	Control Food	1%	2%	4%
Moisture	8.11	8.38	8.50	8.41
Protein, crude	23.81	23.44	23.56	23.63
Fat	13.16	14.27	12.95	13.01
Atwater energy ^1^ (kcal/kg)	3569	3631	3567	3588
Ash	5.81	5.96	5.98	5.85
Crude fiber	2.4	2.3	2.1	1.8
Total dietary fiber	6.1	6.7	6.2	6.8
Total insoluble fiber	4.6	5.1	5.7	5.3
Total soluble fiber	1.5	1.6	0.5	1.5
Calcium	1.02	1.00	1.06	1.04
Phosphorus	0.75	0.77	0.79	0.78
Sodium	0.40	0.40	0.41	0.40
Capric acid (10:0)	<0.02	<0.02	<0.02	<0.02
Lauric acid (12:0)	<0.02	<0.02	<0.02	<0.02
Myristic acid (14:0)	0.07	0.07	0.07	0.07
Palmitic acid (16:0)	2.92	2.79	2.70	2.72
Palmitoleic acid (16:1)	0.70	0.66	0.63	0.64
Steric acid (18:0)	0.86	0.81	0.80	0.80
Oleic acid (18:1)	4.68	4.45	4.33	4.38
Arachidic acid (20:0)	0.02	<0.02	0.02	<0.02
LA (18:2 (n-6))	2.69	2.73	2.71	2.74
aLA (18:3 (n-3))	0.18	0.22	0.27	0.35
ARA (20:4 (n-6))	0.05	0.05	0.05	0.05
EPA (20:5 (n-3))	<0.02	<0.02	<0.02	<0.02
DHA (22:6 (n-3))	<0.02	<0.02	<0.02	<0.02
Lysine	1.10	1.08	1.10	1.09
Threonine	0.89	0.85	0.84	0.85
Methionine	0.53	0.54	0.53	0.54
Cystine	0.31	0.31	0.31	0.31
Tryptophan	0.27	0.27	0.27	0.29

^1^ Calculated from analyticals using modified Atwater numbers (kcal/g of 3.5 for protein, 8.5. for fat, and 3.5 for nitrogen-free extract). aLA, alpha-linolenic acid; ARA, arachidonic acid; DHA, docosahexaenoic acid; EPA, eicosapentaenoic acid; LA, linoleic acid.

**Table 2:** Body weight and selected serum biochemistry parameters at baseline, end of study, and change from baseline (Day 0) in dogs that consumed foods containing 0%, 1%, 2%, or 4% of added fiber-bound polyphenol ingredients.

		Fiber Bundle Percentage in Food		
Parameter	Control Food	1%	2%	4%
Body weight, kg				
Day 0	10.7 ± 0.55	10.6 ± 0.55	10.9 ± 0.55	10.5 ± 0.55
Day 31	10.4 ± 0.56	10.3 ± 0.56	10.8 ± 0.56	10.3 ± 0.56
Change	−0.3 ± 0.10 ^1^	−0.3 ± 0.10 ^1^	−0.1 ± 0.10	−0.1 ± 0.10
Food intake, kcal/(body weight in kg)^0.75^	112 ± 5.5	107 ± 5.5	119 ± 5.5	109 ± 5.5
Albumin, mg/dL				
Day 0	3.57 ± 0.08	3.62 ± 0.08	3.45 ± 0.08	3.51 ± 0.08
Day 31	3.68 ± 0.08	3.64 ± 0.08	3.52 ± 0.08	3.60 ± 0.08
Change	0.12 ± 0.04	0.02 ± 0.04	0.08 ± 0.04	0.09 ± 0.04
Total protein, mg/dL				
Day 0	5.58 ± 0.09	5.78 ± 0.09	5.54 ± 0.09	5.65 ± 0.09
Day 31	5.54 ± 0.11	5.60 ± 0.11	5.47 ± 0.11	5.64 ± 0.11
Change	−0.04 ± 0.06	−0.17 ± 0.06 ^1^	−0.08 ± 0.06	−0.01 ± 0.06
Urea nitrogen, mg/dL				
Day 0	12.2 ± 0.8	13.1 ± 0.8	14.1 ± 0.8	12.5 ± 0.8
Day 31	11.5 ± 0.8	12.9 ± 0.8	13.9 ± 0.8	12.4 ± 0.8
Change	−0.6 ± 0.6	−0.2 ± 0.6	−0.2 ± 0.6	−0.1 ± 0.6
Creatinine, mg/dL				
Day 0	0.71 ± 0.03	0.70 ± 0.03	0.68 ± 0.03	0.72 ± 0.03
Day 31	0.75 ± 0.04	0.71 ± 0.04	0.73 ± 0.04	0.76 ± 0.04
Change	0.03 ± 0.02	0.01 ± 0.02	0.06 ± 0.02 ^1^	0.04 ± 0.02 ^1^
Triglycerides, mg/dL				
Day 0	63.2 ± 5.6	59.1 ± 5.6	64.2 ± 5.6	65.6 ± 5.6
Day 31	66.9 ± 5.1	62.7 ± 5.1	68.7 ± 5.1	61.6 ± 5.1
Change	3.7 ± 4.1	3.7 ± 4.1	4.5 ± 4.1	−4.0 ± 4.1
Cholesterol, mg/dL				
Day 0	195.1 ± 14.2	207.6 ± 14.2	191.2 ± 14.2	219.2 ± 14.2
Day 31	203.5 ± 14.7	211.9 ± 14.7	184.0 ± 14.7	222.4 ± 14.7
Change	8.2 ± 5.8	4.3 ± 5.8	−7.2 ± 5.8	3.2 ± 5.8

Values are least square means ± standard errors. ^1^ Significantly different (*p* < 0.05) from baseline (Day 0).

**Table 3:** Fecal moisture, ammonium, and pH at baseline, end of study, and change from baseline (Day 0) and stool scores in dogs that consumed foods containing 0%, 1%, 2%, or 4% of added fiber-bound polyphenol ingredients.

		Fiber Bundle Percentage in Food		
Parameter	Control Food	1%	2%	4%
Moisture				
Day 0	68.3 ± 0.9	69.8 ± 0.9	68.1 ± 0.9	67.8 ± 0.9
Day 10, % of Day 0	103 ± 1 ^a1^	98 ± 1 ^b^	101 ± 1 ^a,b^	103 ± 1 ^a1^
Day 31, % of Day 0	105 ± 1 ^a1^	100 ± 1 ^b^	104 ± 1 ^a1^	104 ± 1 ^a1^
Ammonium, mmol/g				
Day 0	0.040 ± 0.002	0.044 ± 0.002	0.046 ± 0.002	0.045 ± 0.002
Day 10, % of Day 0	97 ± 9	107 ± 9	94 ± 9	95 ± 9
Day 31, % of Day 0	107 ± 9	110 ± 9	102 ± 9	104 ± 9
pH				
Day 0	5.88 ± 0.06	5.92 ± 0.06	5.90 ± 0.06	5.97 ± 0.06
Day 10, % of Day 0	100 ± 1	100 ± 1	100 ± 1	99 ± 1
Day 31, % of Day 0	99 ± 1	102 ± 1	100 ± 1	98 ± 1
Stool score				
Day 0	4.2 ± 0.20	4.2 ± 0.19	4.5 ± 0.19	4.0 ± 1.19
Day 10	4.3 ± 0.19	4.4 ± 0.19	4.7 ± 0.19	4.5 ± 0.19
Day 31	4.2 ± 0.19	4.4 ± 0.19	4.3 ± 0.19	4.5 ± 0.19

Values are least square means ± standard errors. ^1^ Significantly different (*p* < 0.05) from baseline (Day 0). Different superscripted letters represent significant differences within a row (*p* < 0.05).

**Table 4:** Fecal short-chain fatty acids at baseline (Day 0) and Days 10 and 31 in dogs that consumed foods containing 0%, 1%, 2%, or 4% of added fiber-bound polyphenol ingredients.

		Fiber Bundle Percentage in Food		
SCFA	Control Food	1%	2%	4%
Acetic acid				
Day 0, μg/g	4100 ± 180	4243 ± 173	4744 ± 173	4538 ± 173
Day 10, % of Day 0	114 ± 7	103 ± 7	103 ± 7	104 ± 7
Day 31, % of Day 0	112 ± 7	104 ± 7	93 ± 7	102 ± 7
Propionic acid				
Day 0, μg/g	3229 ± 191	3000 ± 183	3231 ± 183	3239 ± 183
Day 10, % of Day 0	91 ± 9	104 ± 9	102 ± 9	96 ± 9
Day 31, % of Day 0	86 ± 9	92 ± 9	90 ± 9	97 ± 9
Butyric acid				
Day 0, μg/g	3547 ± 449	3960 ± 430	3355 ± 430	3962 ± 430
Day 10, % of Day 0	92 ± 25	102 ± 24	123 ± 24	104 ± 24
Day 31, % of Day 0	120 ± 25	110 ± 24	126 ± 24	158 ± 24 ^1^
Valeric acid				
Day 0, μg/g	530 ± 162	468 ± 155	640 ± 155	559 ± 155
Day 10, % of initial	100 ± 80	166 ± 76	160 ± 76	121 ± 76
Day 31, % of initial	141 ± 80	156 ± 76	189 ± 76	299 ± 76 ^1^
Hexanoic acid				
Day 0, μg/g	27 ± 9.8	8 ± 9.5	30 ± 9.5	26 ± 9.5
Day 10, % of Day 0	151 ± 251	316 ± 240	99 ± 240	107 ± 240
Day 31, % of Day 0	120 ± 251	140 ± 240	167 ± 240	756 ± 240 ^1^
2-methylpropionic acid				
Day 0, μg/g	188 ± 21	215 ± 20	257 ± 20	253 ± 20
Day 10, % of Day 0	108 ± 12	117 ± 12	95 ± 12	91 ± 12
Day 31, % of Day 0	120 ± 12	115 ± 12	97 ± 12	98 ± 12
2-methylbutyric acid				
Day 0, μg/g	137 ± 14	151 ± 14	181 ± 14	175 ± 14
Day 10, % of Day 0	97 ± 11	113 ± 11	94 ± 11	91 ± 11
Day 31, % of Day 0	107 ± 11	115 ± 11	92 ± 11	102 ± 11
3-methylbutyric acid				
Day 0, μg/g	223 ± 23	229 ± 22	274 ± 22	275 ± 22
Day 10, % of Day 0	99 ± 12	119 ± 12	102 ± 12	85 ± 12
Day 31, % of Day 0	103 ± 12	109 ± 12	97 ± 12	102 ± 12

^1^ Significantly different (*p* < 0.05) from baseline (Day 0).

**Table 5:** Change from initial concentrations at Days 10 and 31 (natural log day 10 or 31—natural log Day 0) of selected fecal metabolites in dogs that consumed foods containing 0%, 1%, 2%, or 4% of added fiber-bound polyphenol ingredients.

		Fiber Bundle Percentage in Food		
Metabolite	Control Food	1%	2%	4%
Hesperidin				
Day 10 ratio	0.42 ± 0.31 ^a^	2.15 ± 0.31 ^b1^	3.63 ± 0.31 ^c1^	4.55 ± 0.31 ^d1^
Day 31 ratio	0.95 ± 0.31 ^a^	1.85 ± 0.31 ^b1^	3.74 ± 0.31 ^c1^	4.37 ± 0.31 ^c1^
Hesperetin				
Day 10 ratio	0.62 ± 0.38 ^a^	3.59 ± 0.37 ^b1^	4.69 ± 0.37 ^c1^	5.39 ± 0.37 ^c1^
Day 31 ratio	0.67 ± 0.38 ^a^	3.22 ± 0.37 ^b1^	4.33 ± 0.37 ^c1^	5.24 ± 0.37 ^c1^
Ponciretin				
Day 10 ratio	0.53 ± 0.38 ^a^	3.19 ± 0.36 ^b1^	3.93 ± 0.36 ^b1^	4.76 ± 0.36 ^c1^
Day 31 ratio	0.51 ± 0.38 ^a^	2.82 ± 0.36 ^b1^	3.56 ± 0.36 ^b1^	4.75 ± 0.36 ^c1^
Secoisolariciresinol diglucoside				
Day 10 ratio	0.01 ± 0.24 ^a^	1.41 ± 0.23 ^b1^	1.85 ± 0.23 ^b,c1^	2.31 ± 0.23 ^c1^
Day 31 ratio	0.43 ± 0.24 ^a^	0.66 ± 0.23 ^a1^	1.42 ± 0.23 ^b1^	1.83 ± 0.23 ^b1^
Secoisolariciresinol				
Day 10 ratio	0.28 ± 0.26 ^a^	0.87 ± 0.25 ^b1^	1.88 ± 0.25 ^c1^	2.06 ± 0.25 ^c1^
Day 31 ratio	0.50 ± 0.26 ^a^	0.62 ± 0.25 ^a,b1^	1.17 ± 0.25 ^b,c1^	1.80 ± 0.25 ^c1^
Enterodiol				
Day 10 ratio	−0.47 ± 0.30 ^a^	0.91 ± 0.29 ^b1^	0.99 ± 0.29 ^b1^	1.33 ± 0.29 ^b1^
Day 31 ratio	0.32 ± 0.30 ^a^	0.97 ± 0.29 ^b1^	1.00 ± 0.29 ^b1^	2.07 ± 0.29 ^c1^
Arabinose				
Day 10 ratio	0.50 ± 0.15 ^a1^	0.14 ± 0.14 ^b^	0.22 ± 0.14 ^a,b^	0.56 ± 0.14 ^a1^
Day 31 ratio	0.25 ± 0.15 ^a^	−0.26 ± 0.14 ^b^	−0.23 ± 0.14 ^b^	0.30 ± 0.14 ^a1^
Ribulose/xylulose				
Day 10 ratio	0.12 ± 0.11	0.01 ± 0.15	0.22 ± 0.14	0.35 ± 0.14 ^1^
Day 31 ratio	0.17 ± 0.11	−0.01 ± 0.15	−0.10 ± 0.14	0.17 ± 0.14

^1^ Significantly different (*p* < 0.05) from baseline (Day 0). Different superscripted letters represent significant differences within a row (*p* < 0.05).

**Table 6:** PERMANOVA analysis of bacterial taxa and KEGG pathways in feces from dogs that consumed the study foods.

Microbiome	Food Comparison	Mean Squares	*p*-Value Corrected *	*p* Value
**Taxa**				
Phylum	All	0.025	0.929	0.888
Family	All	0.099	0.990	0.923
Genus	All	0.131	0.981	0.969
**KEGG pathways**				
Arginine	All	0.0000009	0.989	0.916
Benzoate	All	0.0000000	0.989	0.983
Butyrate	All	0.0000025	0.989	0.976
Carbohydrate-active enzymes	All	0.0000018	0.989	0.971
Phenylalanine	All	0.0000002	0.989	0.912
Propionate	All	0.0000016	0.989	0.928
Tryptophan	All	0.0000000	0.989	0.946
Tyrosine	All	0.0000002	0.989	0.903

* By Benjamini–Hochberg method.

## Data Availability

Data are available in the paper and its supplemental files or by request from the corresponding author.

## Supplementary Materials

The following supporting information can be downloaded at: https://www.mdpi.com/article/10.3390/ani12050627/s1, Table S1: Formulations of the foods used in this study.
