# Supplementary material for: Feeding Fiber-Bound Polyphenol Ingredients at Different Levels Modulates Colonic Postbiotics to Improve Gut Health in Dogs
Source: Animals (Basel). 2022 Mar 2;12(5):627. doi: 10.3390/ani12050627 (PMC8909809; doi:10.3390/ani12050627)
Supplement: Supplementary file 1 [file animals-12-00627-s001.zip › animals-1596852-supplementary.pdf]

Table S1. Formulations of the foods used in this study.

	Fiber bundle percentage in food			
**Ingredients, %**	**Control**	**1%**	**2%**	**4%**
Rice, brewers	30	29	29	29
Corn, yellow, whole	15	15	15	15
Corn, gluten, meal	10	10	10	9.9
Chicken meal	10.1	10	10	9.9
Barley, pearled, cracked	9.8	9.8	9.7	9.6
Chicken fat, refined	7.0	6.9	6.8	6.7
Chicken, dried, 10% ash	5	5	5	5
Palatability enhancer, C17007	3.5	3.5	3.5	3.5
Pecan shells, ground	0	0.43	0.85	1.7
Beet, pulp, pelleted	0	0.18	0.36	0.71
Citrus pulp, dried ground	0	0.15	0.3	0.6
Flax seed, whole brown	0	0.18	0.37	0.74
Cranberry pomace	0	0.06	0.12	0.24
Cellulose, pelleted	2.4	1.8	1.2	0
Palatant, ITE2, dry	1.5	1.5	1.5	1.5
Lactic acid, blend, 84%	1.2	1.2	1.2	1.2
Soybean oil, refined, naturally preserved	1.1	1.1	1.1	1.1
Potassium chloride	1.1	1.1	1.1	1.1
Sodium chloride, iodized	0.6	0.6	0.6	0.6
Choline chloride, liquid, 70%	0.45	0.45	0.45	0.45
Dicalcium phosphate	0.45	0.45	0.45	0.45
Calcium sulfate	0.35	0.35	0.35	0.35
Carnitine, l, 10%	0.3	0.3	0.3	0.3
Vitamin premix	0.21	0.2	0.2	0.2
Glyceryl monostearate	0.2	0.2	0.2	0.2
Vitamin E, adsorbate, 50%	0.13	0.13	0.13	0.13
Taurine	0.12	0.12	0.12	0.12
Mineral, premix, 2305	0.08	0.08	0.08	0.08
Tryptophan, l	0.05	0.05	0.05	0.05
Magnesium oxide	0.05	0.05	0.05	0
